# Exploring accessibility of pretreated poplar cell walls by measuring dynamics of fluorescent probes

**DOI:** 10.1186/s13068-017-0704-5

**Published:** 2017-01-14

**Authors:** Gabriel Paës, Anouck Habrant, Jordane Ossemond, Brigitte Chabbert

**Affiliations:** FARE laboratory, INRA, Université de Reims Champagne-Ardenne, 51100 Reims, France

**Keywords:** Confocal fluorescence microscopy, Pretreatment, Poplar, Accessibility, FRAP

## Abstract

**Background:**

The lignocellulosic cell wall network is resistant to enzymatic degradation due to the complex chemical and structural features. Pretreatments are thus commonly used to overcome natural recalcitrance of lignocellulose. Characterization of their impact on architecture requires combinatory approaches. However, the accessibility of the lignocellulosic cell walls still needs further insights to provide relevant information.

**Results:**

Poplar specimens were pretreated using different conditions. Chemical, spectral, microscopic and immunolabeling analysis revealed that poplar cell walls were more altered by sodium chlorite-acetic acid and hydrothermal pretreatments but weakly modified by soaking in aqueous ammonium. In order to evaluate the accessibility of the pretreated poplar samples, two fluorescent probes (rhodamine B-isothiocyanate–dextrans of 20 and 70 kDa) were selected, and their mobility was measured by using the fluorescence recovery after photobleaching (FRAP) technique in a full factorial experiment. The mobility of the probes was dependent on the pretreatment type, the cell wall localization (secondary cell wall and cell corner middle lamella) and the probe size. Overall, combinatory analysis of pretreated poplar samples showed that even the partial removal of hemicellulose contributed to facilitate the accessibility to the fluorescent probes. On the contrary, nearly complete removal of lignin was detrimental to accessibility due to the possible cellulose–hemicellulose collapse.

**Conclusions:**

Evaluation of plant cell wall accessibility through FRAP measurement brings further insights into the impact of physicochemical pretreatments on lignocellulosic samples in combination with chemical and histochemical analysis. This technique thus represents a relevant approach to better understand the effect of pretreatments on lignocellulose architecture, while considering different limitations as non-specific interactions and enzyme efficiency.

## Background

Lignocellulosic biomass is the only renewable source of fuels, chemicals and materials that can help limiting the impact of climate changes and fossil carbon dependency [1]. Actually, enzymatic bioconversion and upgrading of lignocellulose offers an alternative strategy for the development of environmental-friendly fractionation of plant biomass. Notably, many projects are focused on the biochemical conversion of lignocellulose into fermentable sugars for the production of second generation ethanol and other biofuels [2, 3]. However, the tight association of the plant cell wall constituents, namely cellulose, hemicellulose and lignin, creates a complex network [4] resistant to enzymatic degradation [5] thus representing an important barrier to efficient and economic bioconversion of lignocellulose [2]. Two general classes of factors limit the efficiency of enzymes: (i) structural factors, mainly related to substrate accessibility and depending on lignocellulose heterogeneous porosity [6–9]; (ii) biochemical factors regarding non-specific binding interactions of enzymes onto lignin [10–12], and all the inactivation/inhibition processes [13, 14]. Pretreatments are thus needed to overcome cell wall recalcitrance, to enhance enzyme accessibility and hydrolysis, by removing some components and/or disrupting the plant cell wall network [15]. As a consequence, understanding the features controlling the accessibility of enzymes into pretreated lignocellulose is an important challenge for optimizing biomass transformation processes.

Penetration and progression of enzymes into lignocellulose substrates face several limitations, from tissular to molecular scales. Chemical and structural features limiting enzyme progression in plant cell walls are complex and generally require a multiscale visualization combined to physicochemical characterization to get insights into the changes due to pretreatment [16–18]. Besides chemical and structural characterization of the whole sample, microscopic approaches such as optical microscopy, microspectrophotometry, atomic force microscopy and electron microscopy have provided critical information related to the cell wall modifications caused by both pretreatments and enzyme hydrolysis [19, 20]. Altogether these studies have shown that pretreatments induce the degradation and/or removal of the hemicellulose and lignin, and overall lignocellulose architecture and porosity can be changed drastically [6, 16, 21, 22].

Among microscopy approaches, fluorescence confocal microscopy can yield key insights into plant cell walls: mapping of plant cell walls by using plant cell wall autofluorescence [23]; identification of chemical features by immunolabeling [24, 25] and by measuring the binding properties of fluorescently labelled lignocellulose-active enzymes [8]. Even spatial and temporal imaging of enzymes distribution within complex lignocellulose substrate can be carried out, indicating that cellulases preferentially bind altered plant cell walls [26, 27].

But there is still a lack of a method to characterize the mobility of enzymes into the complex lignocellulosic cell wall network. Usually, various porosimetry methods are applied to give access to the nano- and microarchitecture of lignocellulose [6]. But they are restricted to some physical information (pore size and morphology) and suffer from drawbacks due to sample preparation [9, 28]. Alternatively, interesting attempts using confocal microscopy have investigated the porous structure of cellulose fibres [29]. Other biochemical techniques can provide information on the accessible surface of a specific polymer-like lignin for example [30, 31].

Mobility of various fluorescent probes has been previously evaluated by using fluorescence recovery after photobleaching (FRAP) technique, in cell wall polysaccharides [32–34] and in bioinspired plant cell wall assemblies [35–38]. In addition, fluorescent probes [39] were recently shown to provide a more comprehensive view of the nanoporosity of lignified cell walls [40]. Here, for the first time, we have used the FRAP technique to give an overview of the accessibility of lignocellulose sample, depending on the pretreatment applied, the probe size and the cell wall localization, in combination to physicochemical characterization of the pretreated samples.

## Results and discussion

### Chemical changes induced by pretreatments

**Fig. 1:**
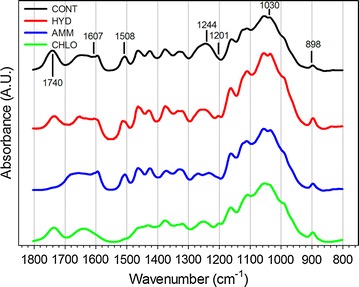
FTIR spectra of untreated (CONT) and pretreated (HYD, AMM and CHLO) poplar samples

Weight losses of 27, 17 and 30% after HYD, AMM and CHLO pretreatments were observed, respectively. Chemical changes were evidenced by FTIR spectroscopy (Fig. 1) and wet chemistry (Table 1). Comparison of IR spectra recorded on pretreated samples vs untreated samples showed some differences depending on pretreatment. CHLO and AMM pretreated samples were the most altered as shown by the strong reduction and/or disappearance of several bands. In CHLO samples, the intensity of the bands at 1607 and 1508 cm^−1^ corresponding to aromatic skeletal vibration [41] was strongly reduced. In AMM samples, the bands at 1740 and 1244 cm^−1^ were reduced, indicating the removal of acetyl-ester of xylan and C–O vibration from guaiacyl units [41]. Compared to CHLO and AMM, IR spectral changes in HYD samples were much lower. Only the smaller intensity of the 1740 cm^−1^ band corresponding to the carbonyl stretching could suggest that non-cellulosic polysaccharide might have been affected. This slight decrease was also observed in CHLO poplar. In addition, compared to the intensity of one main vibration in the region analysed (1030 cm^−1^), the OH plane deformation at 1201 cm^−1^ and the C–H deformation peak of cellulose at 898 cm^−1^ were relatively increased for both CHLO and HYD samples, suggesting an increase of cellulose and/or modification in the cellulose organization. Thus, these pretreatments might have removed some non-cellulosic components from poplar cell walls.

**Table 1:** Composition of untreated and pretreated poplar samples

	CONT	HYD	AMM	CHLO
Lignin content^a^	28.12 ± 0.28	31.83 ± 0.86	27.24 ± 0.14	8.54 ± 0.37
S + G^b^	1864.47 ± 81.76	1273.19 ± 186.77	1727.02 ± 212.27	180.36 ± 19.41
S/G	1.5	1.6	1.6	1.1
Total sugars^a^	56.53 ± 2.23	59.85 ± 2.64	62.14 ± 2.97	65.85 ± 3.33
Glucose^a^	35.95 ± 1.40	44.64 ± 1.86	40.50 ± 1.96	42.32 ± 2.26
Xylose^a^	14.79 ± 0.47	12.25 ± 0.61	15.68 ± 0.72	17.80 ± 0.87
Mannose^a^	2.61 ± 0.11	1.93 ± 0.12	2.97 ± 0.14	3.10 ± 0.10
Arabinose^a^	0.35 ± 0.01	0.07 ± 0.01	0.37 ± 0.01	0.22 ± 0.01
Galactose^a^	0.92 ± 0.03	0.25 ± 0.01	0.89 ± 0.03	0.66 ± 0.02
Rhamnose^a^	0.38 ± 0.01	0,16 ± 0.01	0,37 ± 0.04	0,37 ± 0.01
Galacturonic acid^a^	1.37 ± 0.02	0.50 ± 0.03	1.27 ± 0.07	1.25 ± 0.05
Glucuronic acid^a^	0.16 ± 0.08	0.04 ± 0.01	0.09 ± 0.01	0.13 ± 0.01

^a^Lignin and sugar content expressed as percentage of dry matter

^b^Total yields of syringyl and guaiacyl units released by thioacidolysis expressed as µmol/g lignin

In agreement with IR data, results from composition analysis indicate a strong decrease of the lignin content in CHLO samples, whereas it was slightly modified in AMM and HYD samples (Table 1). Based on the dry matter weight loss, removal of lignin represented 75% of initial lignin in CHLO samples, only 10 and 17% in AMM and HYD, respectively. Consequently, the proportion of polysaccharides increased in pretreated samples, especially in CHLO samples which had the highest sugar content. Main changes in the proportion of hemicellulose (xylose, mannose, glucuronic acid) and pectin monosaccharides (arabinose, galactose, rhamnose, galacturonic acid) occurred in HYD samples. This result is directly related to the autohydrolysis of hemicellulose and pectins by acetic acid and other organic acids due to the cleavage of *O*-acetyl and uronic acid under liquid hot water pretreatments [42–45]. Thus HYD pretreatment removed 35% xylose, whereas less than 10% was lost during AMM and CHLO pretreatments, as previously reported for chlorite-delignified poplar [46]. Removal in minor non-cellulosic sugars such as arabinose, galactose and uronic sugars was even higher than for xylose with 70–85% loss in HYD samples. Interestingly, a smaller fraction of these minor non-cellulosic sugars was also solubilized in both CHLO samples (30–50%) and AMM samples albeit to a much lower degree (10–20%).

Besides these changes in cell wall composition, structural modifications of the residual lignins were also observed. Lower content of lignin monomers (S + G) was recovered after thioacidolysis of both HYD and CHLO samples, whereas AMM samples were not affected, indicating a decrease in labile aryl ether linkages (non-condensed) lignin bonds. In CHLO samples, S + G, expressed as µmol/g lignin, was divided by 10: residual lignin has become highly condensed after CHLO pretreatment. Decrease from 1.5 to 1.1 of the S/G molar ratio further suggests a higher extractability of syringyl groups relatively to guaiacyl groups. Consistently, aryl ether linkages which are predominantly involved in syringyl-rich lignin types are less resistant to chemical degradation than guaiacyl-rich lignin [47, 48]. In HYD samples, the decrease of the non-condensed lignin proportion is in agreement with previous studies showing that during hot water treatment, lignin undergoes both depolymerization and recondensation mechanisms giving rise to pseudo-lignin [20, 43, 49]. Overall, IR and chemical data indicate that AMM samples were very moderately affected; hemicellulose were removed and lignin was modified in HYD samples; lignin was largely removed in CHLO samples.

### Histochemical changes induced by pretreatments

**Fig. 2:**
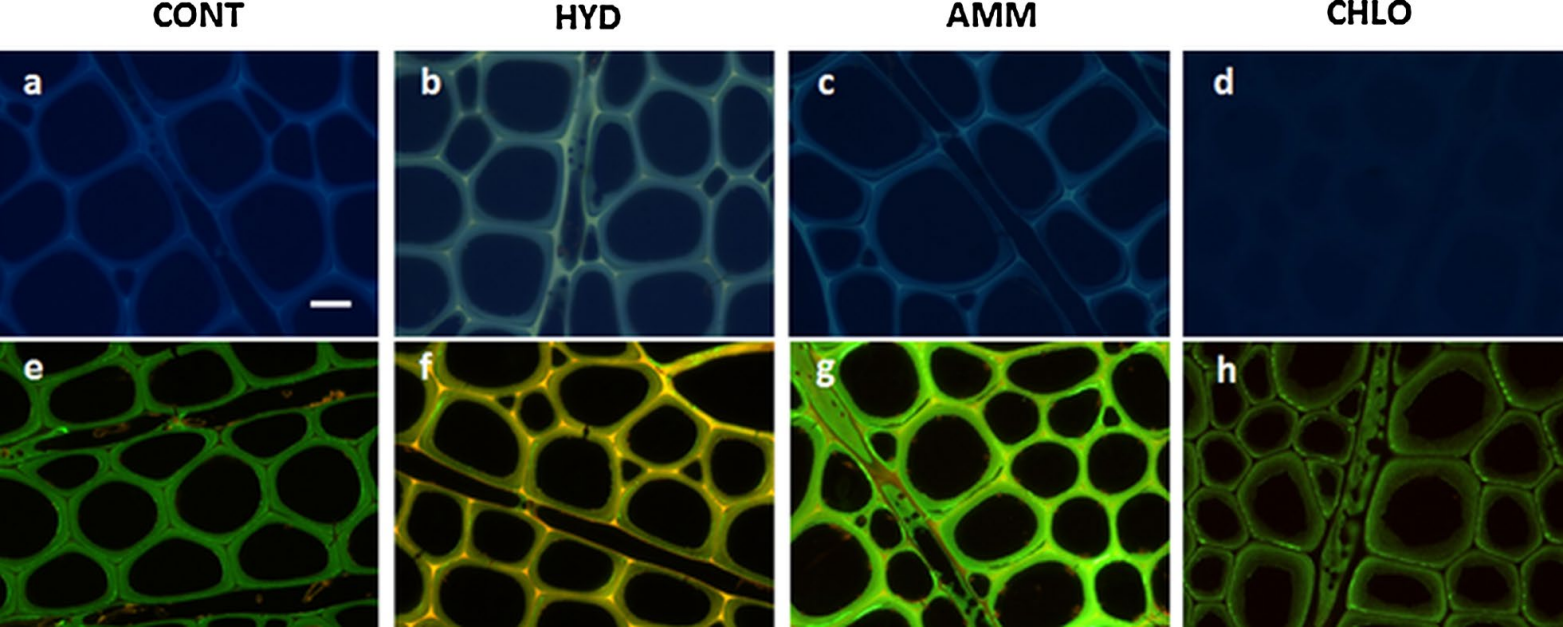
UV autofluorescence (first row **a**–**d**) and xylan immunolabeling (second row **e**–**h**) of poplar sections from control sample (CONT: **a**, **e**) and pretreated samples (HYD: **b**, **f**; AMM: **c**, **g**; CHLO: **d**, **h**)

UV autofluorescence in poplar cell walls is mainly due to lignin and is dependent on the lignin concentration and monolignols chemical arrangement within the cell walls [50]. Microscopic observations of cross sections of the poplar samples (mature xylem) show that the strong autofluorescence in the untreated samples (Fig. 2a) has almost disappeared in CHLO samples (Fig. 2d). HYD samples display altered UV fluorescence (Fig. 2b), whereas AMM samples (Fig. 2c) show a slight decrease of the intensity of fluorescence in the secondary walls. Change in UV autofluorescence intensity is thus consistent to previously measured lignin content.

The distribution of xylan was further investigated using the LM10 monoclonal antibody which reacts specifically with low substituted and unsubstituted β-(1-4)-linked xylose residues as in glucuronoxylan [51]. In the untreated sample, xylan labelling was detected as green fluorescence in all the xylem cells with stronger labelling in the outer secondary cell walls but almost no labelling in the cell corner middle lamella (Fig. 2e). This distribution of xylan is similar to previous observations of poplar xylem [25, 52]. In HYD samples, xylan labelling was faint in the secondary cell walls of the fibres (and vessels) (Fig. 2f). The labelling pattern in AMM samples was similar albeit slightly higher compared to the untreated sample (Fig. 2g). In CHLO samples, xylan immunofluorescence was decreased and was observed mainly in the outer layers of the secondary walls (Fig. 2h). Changes in the pattern of xylan immunolabeling may result from the effect of pretreatment on the number of epitopes and/or their accessibility to immunoprobe [53]. Nevertheless, observations of HYD samples suggesting xylan removal are consistent with chemical data (Table 1) and with the recent studies investigating the impact of hot water pretreatment on xylan removal from cell walls using immune-gold labelling at ultrastructural levels [25]. Overall, chemical and microscopic analyses reveal that poplar cell walls are the most altered by CHLO and HYD pretreatments.

### Cell wall accessibility in pretreated samples

To complement the characterization of the pretreated samples, accessibility of poplar cell walls was evaluated at the molecular scale using some fluorescent dextran-based probes, whose mobility was determined by the FRAP technique. Indeed, such probes are considered as having few or no interaction with plant cell wall polymers [35, 36] and their size and low dispersity (Table 2) are in the range of typical enzymes degrading plant cell walls like cellulases and xylanases [38, 54, 55]. Rhodamine B was chosen as the fluorophore appended to the dextran probe since rhodamine B is excited beyond 540 nm, which is quite far from the maximum lignin excitation that is around 350–400 nm [23]. First, experimental conditions had to be set up to determine several appropriate parameters: probe concentration, buffer pH, poplar sample/probe incubation time and optimal microscopic parameters. Based on these trials (data not shown), incubation of the poplar sections was performed in 0.02 M DXR20 or DXR70 probe in citrate–phosphate buffer at pH 5.0 for 24 h. Excitation at 543 nm was used for both probes (Table 2), and microscope detector gain was finely tuned so that the fluorescence measured was only emitted by the DXR probes and not by the plant cell wall autofluorescence.

**Table 2:** Main properties of fluorescent probes

	Theoretical MW (kDa)	Measured MW (kDa)	Dispersity index	Measured *R*_H_ (nm)	Maximum excitation/emission *λ* (nm)
DXR20	20.0	20.9 ± 0.4	1.4	3.1	541/572
DXR70	70.0	70.0 ± 1.2	1.5	5.8	541/572

**Fig. 3:**
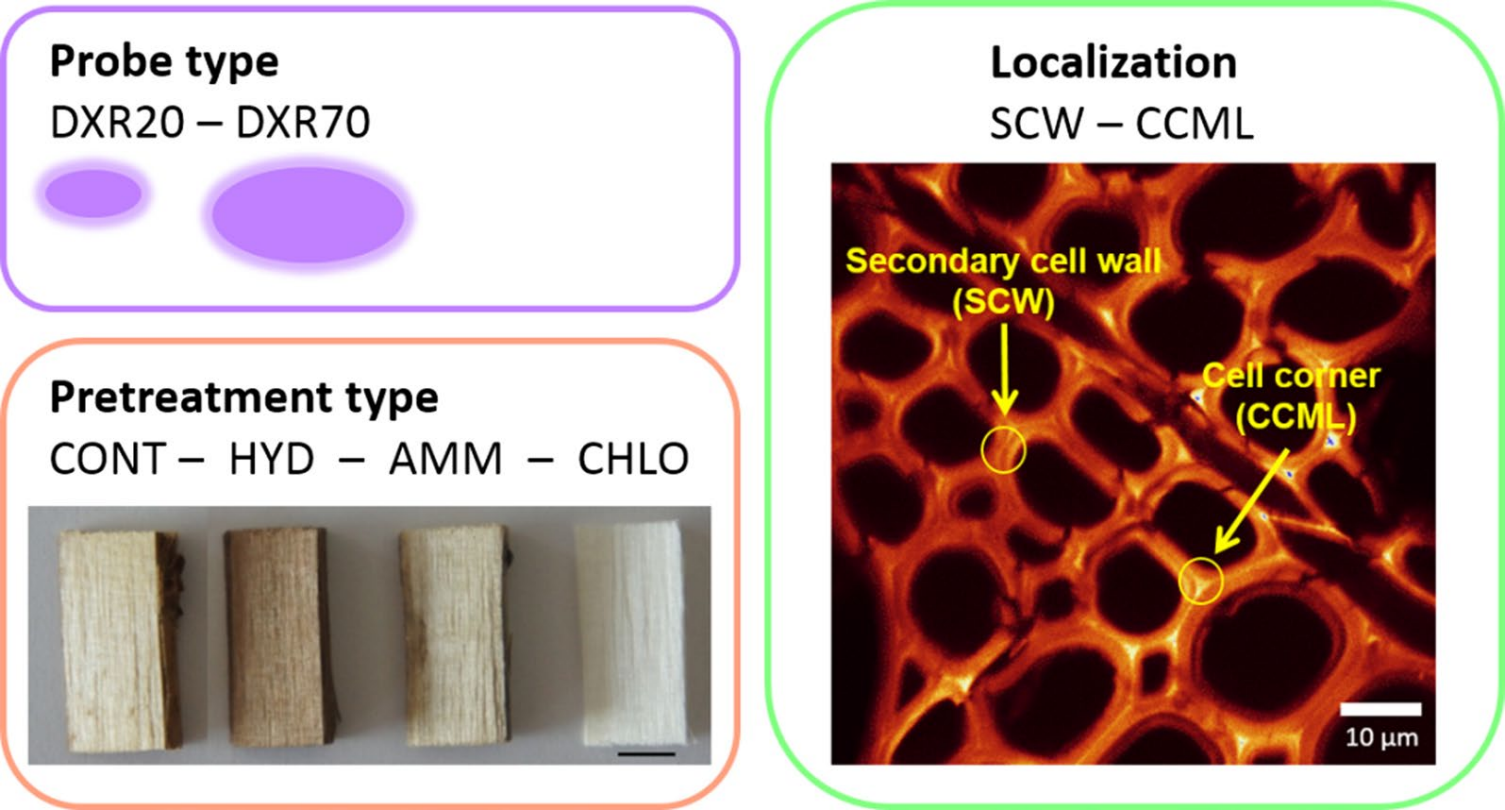
Parameters modulated for the probe mobility measurements and their different levels. Probe type: DXR20 and DXR70 (two levels); pretreatment type: CONT, HYD, AMM and CHLO (four levels); localization: SCW and CCML (two levels)

In order to perform a relevant analysis, mobility experiments were organized as a full factorial experiment in which 3 parameters were varied on at least 2 levels (Fig. 3): the fluorescent probe size (20 kDa for DXR20 and 70 kDa for DXR70), the pretreatment type (CONT, HYD, AMM and CHLO) and the cell wall localization (secondary cell walls, SCW and cell corner middle lamella, CCML). The effect of each factor was determined regarding the 2 responses obtained from the mobility measurements calculated by FRAP: the probe diffusion coefficient *D*, which is the surface the probe can move in one second and is related to the structural and chemical features of both the probe and its environment which can impact probe mobility; the probe mobile fraction *MF*, which is more directly related to the accessibility of the probe, thus to the structural features.

**Fig. 4:**
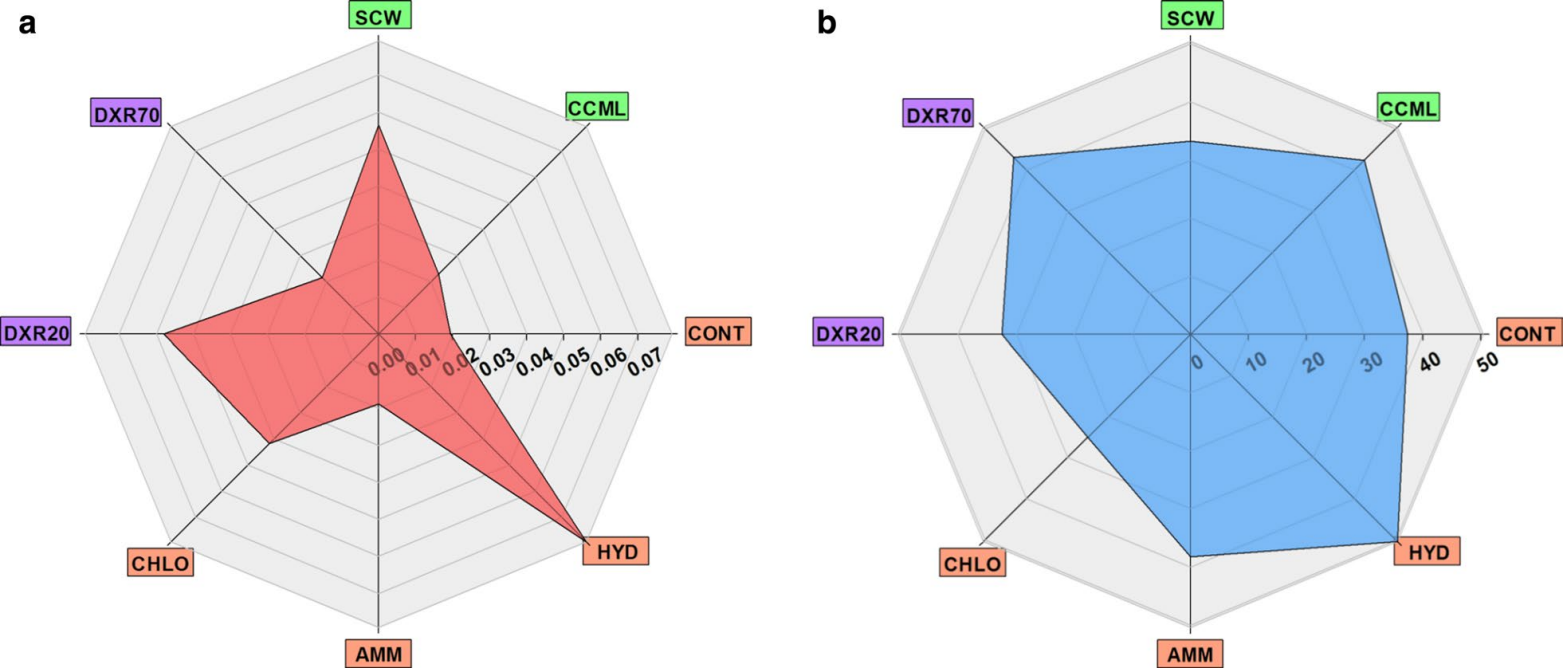
**a** Averaged diffusion coefficient, *D* and **b** mobile fraction, *MF,* values of each level for each parameter. Localization parameter (SCW and CCML) is in green, pretreatment parameter (CONT, HYD, AMM and CHLO) in orange and probe type (DXR20 and DXR70) parameter in purple. *D* is expressed in µm^2^ s^−1^, *MF *in %

First, averaged values of each level for each factor were calculated for *D* and *MF* (Fig. 4). Regarding cell wall localization, *D* is more than twice faster in SCW than in CCML, whereas *MF* in CCML (42%) is significantly higher than in SCW (33%) (Fig. 4). Type of pretreatment shows some contrasted results: the highest *D* value is obtained for HYD, the lowest for CONT and AMM, while CHLO is in-between. For the *MF*, HYD also reaches the maximum value (nearly 50%), CONT and AMM are just below (40%) but CHLO is much lower (25%) (Fig. 4b). Probe type factor indicates that DXR20 diffusion is more than twice higher than that of DXR70 but the *MF *of the latter is higher (43% vs 32%).

**Fig. 5:**
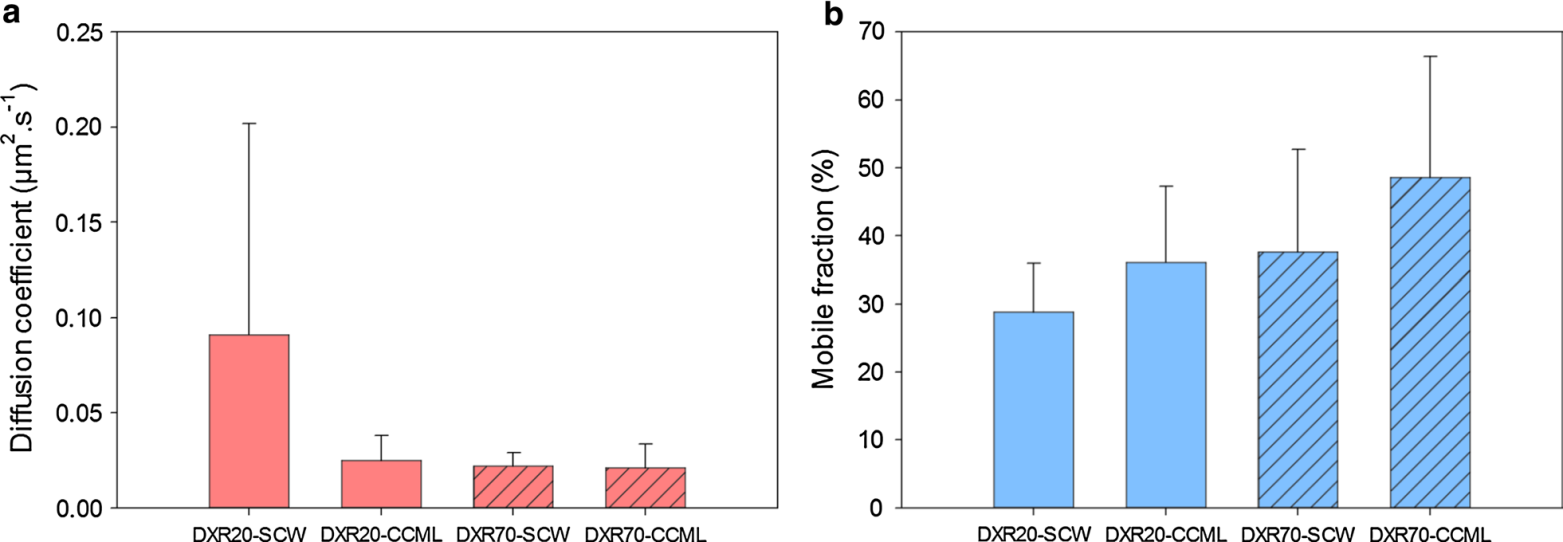
**a** Averaged diffusion coefficient, *D* and **b** mobile fraction, *MF,* values for the pretreatment parameter depending on probe type (DXR20 and DXR70) and localization parameters (SCW and CCML)

**Fig. 6:**
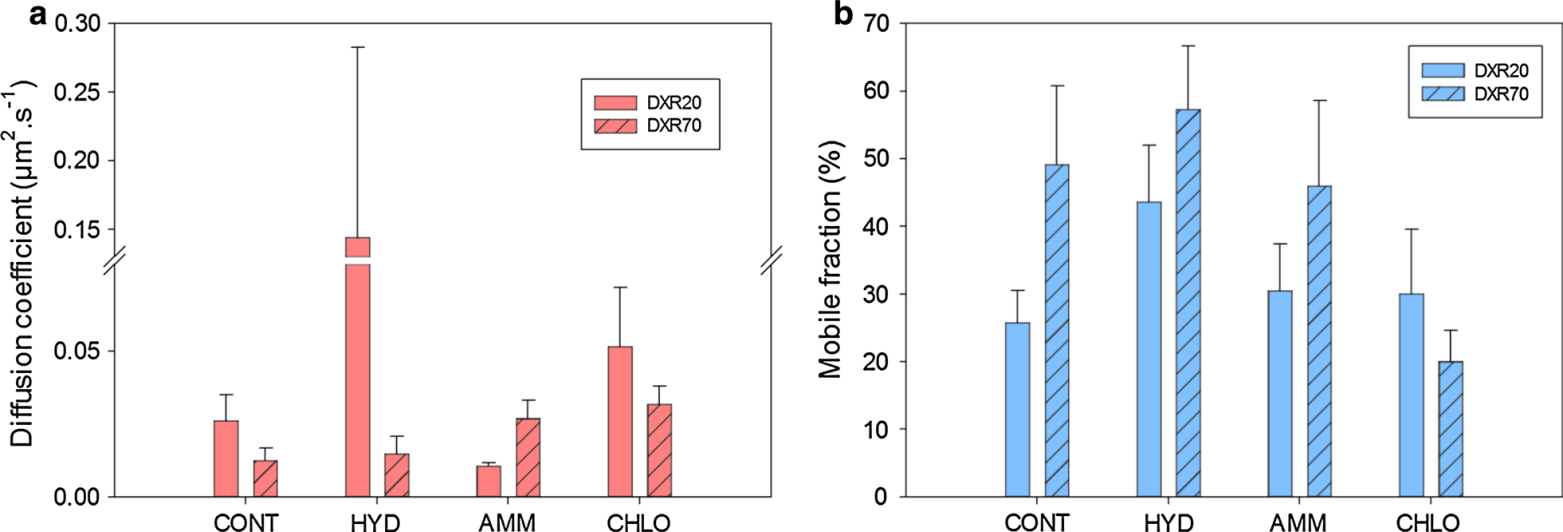
**a** Averaged diffusion coefficient, *D* and **b** mobile fraction, *MF*, values for the localization parameter depending on probe type (DXR20 and DXR70) and pretreatment parameters (CONT, HYD, AMM and CHLO)

These results give some general trends regarding the impact of each factor, but since they are based on averaged values, they mask some discrepancies and the effect of combined factors cannot be described. So in order to provide a better interpretation of the data, only the effect of pretreatment was averaged so that the effect of probe type and localization could be compared (Fig. 5). Clearly, *D* is much higher for DXR20 in SCW than in CCML, while DXR70 diffusion is not influenced by the localisation (Fig. 5a). But given the high standard-deviation for DXR20-SCW, this means that there must be some large differences depending on pretreatment type. *MF* was shown previously to be higher for DXR70 than for DXR20: this difference originates from the localization, since for both probes, *MF* is higher in CCML than in SCW (Fig. 5b). In order to investigate the role of pretreatment, the effect of localization was averaged so that the effect of probe type and pretreatment could be compared (Fig. 6). Diffusion of DXR20 is higher than that of DXR70 in all pretreated samples, except for AMM samples (Fig. 6a). Importantly, diffusion in HYD samples is nearly 10-times faster for DXR20 than for DXR70. So DXR20 reaches a very high diffusion when, simultaneously, measurement is performed in SCW of HYD samples. Contrarily, *MF *(Fig. 6b) is increased for DXR70 compared to DXR20 in all pretreatments except for CHLO.

### Correlation between accessibility and chemical properties of pretreated samples

The determination of the accessibility of two different probes in two different cellular localizations from three differently pretreated poplar samples can help understand the impact of pretreatment in combination with chemical and histochemical analysis of the samples, in comparison to control samples.

In AMM samples, accessibility of the probes does not differ very much from the control on average (Fig. 4). The only striking difference refers to probe size (Fig. 6): diffusion and mobile fractions of DXR20 are lower than those of DXR70 in both CCML and SCW, which can seem counterintuitive, because a smaller probe is expected to diffuse more slowly, in the case only structural effects are expected to control diffusion. So slowed down diffusion of DXR20 might be explained by some biochemical interactions occurring between the probe and some chemical motifs appearing in AMM samples and to be related to the loss of acetyl groups and/or to the weak modifications in hemicellulose, together with slight reduction in lignin content. These interactions would occur in the nanopores existing in the AMM samples which DXR20 can reach. For HYD samples, the large increase of accessibility (Fig. 4) is directly related to both lignin modification and to the removal of hemicellulose and of xylan in particular. For example, the lower xylan content shown by both chemical and histochemical analysis seems to facilitate the diffusion of DXR20 probe, but not that of DXR70, but *MF *is higher for DXR70. This can be interpreted as a sieving effect in the cell wall porosity: a smaller probe can access and diffuse in smaller pores, while a twice bigger probe is excluded from these pores so it can access a larger area. This analysis thus demonstrates that HYD pretreatment affects cell wall porosity to a larger extent than chlorite delignification does. This observation is in good agreement with studies reporting that hydrothermal pretreatments increase porosity and accessible surface [22, 56].

Accessibility of probes in CHLO samples does not reach that measured in HYD samples, whereas lignin removal is much higher. Moreover, even if diffusion in CHLO samples is better than in CONT samples, *MF* is the lowest among all samples analysed. CHLO pretreatment is known to have a dual effect: large removal of lignin thus drastically modifying the interactions between cellulose and hemicellulose and the formation of highly condensed lignin likely altering lignin–carbohydrate complex (LCC) bonds between hemicellulose and residual lignin. Several studies have shown that partial lignin removal rather than complete delignification combined with xylan removal would be more efficient to increase cell wall accessibility [56, 57]. Consequently, removing lignin in CHLO samples might induce rearrangement of the xylan matrix between cellulose fibrils thereby altering nanoporosity of the cell walls and probe accessibility [21].

## Conclusions

Within the context of biorefinery, understanding the factors which control enzyme hydrolysis is essential. Here for the first time, the FRAP technique has been used to investigate one of these factors, the accessibility, by measuring the mobility into the lignocellulose cell walls of molecular probes whose size is representative of enzymes degrading plant materials. Overall, FRAP can report various types of information: structural accessibility of different probes, allowing to finely measure some threshold effects, in complement to porosimetry techniques; biochemical interactions by using probes interacting with cell wall chemical motifs [38, 58]; these structural and biochemical data can be evaluated at the cellular scale, in addition to histochemical analysis which can pinpoint many different types of chemical motifs [24, 25].

Combinatory analysis of pretreated poplar samples has demonstrated that even the partial removal of hemicellulose in poplar cell walls contributes to facilitate the accessibility to dextran molecular probes. Nearly complete removal of lignin is detrimental for accessibility probably because cellulose and hemicellulose collapse. On a structural point of view, evaluation of accessibility through the measurement of accessibility by FRAP is a relevant approach to better understand and select the impact of pretreatment.

Here, only accessibility of some molecular probes was assayed. Other important parameters such as non-specific interactions of probes should be also analysed and combined to accessibility [40]. These data might be considered in order to evaluate the correlation between structural/biochemical accessibility of enzymes and their catalytic efficiency.

## Methods

### Fluorescent probes

All chemicals used for analysis and pretreatment were of analytical grade. Two fluorescent probes rhodamine B-isothiocyanate–dextrans of 20 and 70 kDa (DXR20 and DXR70, references 73,766 and T1162, respectively) were purchased from Sigma-Aldrich (Saint-Quentin-Fallavier, France). According to provider information, one rhodamine B molecule was bound to the dextran backbone every 100–500 glucose unit.

Absolute molecular weight (MW), MW distribution and hydrodynamic radius (*R*_H_) of the fluorescent probes were determined by SEC–MALS–QELS. To summarize, 150 µL of each probe in 50 mM sodium nitrate buffer was injected at 0.6 mL/min on a KW 802.5 column equilibrated at 30 °C connected to the HPLC system (Waters 717), equipped as follows: degas, UV–visible detector (Waters 2996), multi-angle static light-scattering (MALS) detector DAWN HELEOS II (Wyatt, Santa Barbara, USA), dynamic light-scattering detector DynaPro NanoStar (Wyatt), refraction index detector (Waters 2414). Analysis of the chromatogram was performed with the ASTRA 6.1 software (Wyatt). Properties of the fluorescent probes are summarized in Table 2.

### Poplar sample preparation and chemical pretreatment

Poplar wood samples were collected from 3-year-old short rotation coppice grown in experimental fields in Orléans, France. Small wood blocks (3–4 mm width × 2 cm long) were isolated from the basal region and dried overnight at 40 °C in an air-forced oven.

Three different types of pretreatment were applied in triplicates to small wood blocks. Hydrothermal treatment (HYD) was performed for 1 h at 170 °C at a ratio of 15 mL water/g poplar using mineralization reactors equipped with Teflon tubes (Parr) and an oil bath [19]. Sodium chlorite-acetic acid delignification treatment (CHLO) was performed on 1 g poplar using acetic acid (0.15 mL) and sodium chlorite (1.25 g NaClO_2_) at 70 °C for 1 h; the reaction was repeated 5 times [59]. Soaking in aqueous ammonia (AMM) treatment was carried out as previously described [60]. Poplar was soaked into 33% aqueous ammonium (12 mL/g poplar) at room temperature for 6 days. Control samples (CONT) were also obtained after water washing at 4 °C (1 h). After pretreatment, the samples were washed several times with deionized water until the pH of the wash was about 6.0. Then the samples were dried at 40 °C in an air-forced oven until their weight remained constant.

### Chemical analysis

Control and pretreated samples were grinded to 200 µm size prior to infrared spectral analysis and wet chemical degradation. Fourier transform infrared (FTIR) spectroscopy was carried out with a Nicolet 4600 instrument (Thermo Fischer Scientific, USA). KBr disks containing 2 mg of samples were scanned 16 times from 400 to 4000 cm^−1^ at 4 cm^−1^ resolution while subtracting background spectra measured in the air. FTIR spectra were corrected for baseline and normalized on area spectra from 1900 to 800 cm^−1^.

Wet chemistry analysis consisted in the determination of (i) sugar monomer composition using a two-step sulfuric acid hydrolysis followed by high-performance anion-exchange chromatography (HPAEC) with 2-d-deoxyribose as internal standard, (ii) lignin content using spectrophotometric method after acetyl bromide dissolution of the lignocelluloses and (iii) monomer composition of the alkyl aryl ether lignin structures as performed by thioacidolysis as previously described [19].

### Wide-field fluorescence microscopy

Poplar samples were cut into small fragments (3 × 3 mm) prior to embedding into polyethylene glycol (PEG) resin or EPON resin. Samples were gradually dehydrated using ethanol series then acetone prior to epoxy resin impregnation and embedding (Epoxy Embedding Medium, EEM hardener DDSA and EEM hardener NMA, Fluka, USA). Block specimens were cut into 0.5 µm-thick sections using a microtome (MICROM) for UV autofluorescence observation using an Axioskop epifluorescence microscope equipped with filters at 340 nm for excitation and 430 nm for emission (Zeiss, Germany). Xylan immunolocalization was performed using LM10 and LM11 antibodies (PlantProbes, United Kingdom) which specifically bind linear xylan (LM10) and both linear and highly substituted xylan (LM11) [51]. Immunolabeling was performed as previously described [61] using Alexa Fluor 488 goat anti-rat IgG (H + L) (Life Technologies, USA) as secondary antibody prior to observations by fluorescence microscopy (Zeiss, Germany).

### Confocal laser scanning microscopy (CLSM) and fluorescence recovery after photobleaching (FRAP) analysis

Small poplar fragments (3 × 3 mm) were immersed in graded aqueous PEG solutions (MW 1450 g/mol) up to 100% PEG at 60 °C. Embedding was then accomplished by cooling down PEG mixture to 25 °C. Sections of 60 µm-thickness were obtained from PEG-embedded specimens using disposable microtome blade. PEG was removed from the sections by water washing. Sections were incubated in 50 mM citrate–phosphate buffer at pH 5 containing 0.02 M fluorescent probe DXR20 or DXR70 for 24 h at 20 °C in the dark, then mounted between cover glass and a #1.5H cover-slip glass slide in phosphate buffer. FRAP experiments were performed using a Leica TCS SP2 (Mannheim, Germany) with a 63× oil immersion objective and a numerical aperture of 1.4, equipped with a 543 nm argon laser, in a controlled-temperature room (20 ± 2 °C). Images were collected using the following parameters: 1× zoom factor, 512 × 512 pixels size at a frequency of 400 Hz, one acquisition, with a circular region of interest (ROI) of 4 μm diameter. For FRAP experiments, prebleaching was performed with laser at 20% of its power and acquisition of five reference images, followed by bleaching with laser at 100%, with acquisition of 40 images and finally post-bleaching (laser at 20%) with acquisition of images until the bleached ROI intensity was constant. Calculation of the diffusion *D* and the mobile fraction *MF *were performed as previously described [35, 36].

### Statistical analysis

The effect of 3 factors (probe size, pretreatment type, cell wall localisation) was evaluated using ANOVA analysis and Fisher test using Design Expert 8.0 (Stat-Ease, Minneapolis, USA).

## Abbreviations

CONT: control treatment
HYD: hydrothermal treatment
CHLO: sodium chlorite-acetic acid treatment
AMM: aqueous ammonia treatment
CCML: cell corner middle lamella
SCW: secondary cell wall
DXR20: rhodamine B-isothiocyanate dextran 20 kDa
DXR70: rhodamine B-isothiocyanate dextran 70 kDa
FRAP: fluorescence recovery after photobleaching
D: diffusion coefficient
MF: mobile fraction
G: guaiacyl
S: syringyl
